# Metagenomic pathogen sequencing in resource-scarce settings: Lessons learned and the road ahead

**DOI:** 10.3389/fepid.2022.926695

**Published:** 2022-08-15

**Authors:** Christina Yek, Andrea R. Pacheco, Manu Vanaerschot, Jennifer A. Bohl, Elizabeth Fahsbender, Andrés Aranda-Díaz, Sreyngim Lay, Sophana Chea, Meng Heng Oum, Chanthap Lon, Cristina M. Tato, Jessica E. Manning

**Affiliations:** ^1^Department of Critical Care Medicine, National Institutes of Health Clinical Center, Bethesda, MD, United States; ^2^Laboratory of Malaria and Vector Research, National Institute of Allergy and Infectious Diseases, Rockville, MD, United States; ^3^International Center of Excellence in Research, National Institute of Allergy and Infectious Diseases, Phnom Penh, Cambodia; ^4^Chan Zuckerberg Initiative, Redwood City, CA, United States; ^5^Vaccine Immunology Program, Vaccine Research Center, National Institute of Allergy and Infectious Diseases, Bethesda, MD, United States; ^6^Department of Medicine, University of California, San Francisco, San Francisco, CA, United States

**Keywords:** metagenomics, next-generating sequencing, low- and middle-income countries (LMICs), biosurveillance, COVID-19, genomic surveillance, emerging infectious diseases

## Abstract

Metagenomic next-generation sequencing (mNGS) is the process of sequencing all genetic material in a biological sample. The technique is growing in popularity with myriad applications including outbreak investigation, biosurveillance, and pathogen detection in clinical samples. However, mNGS programs are costly to build and maintain, and additional obstacles faced by low- and middle-income countries (LMICs) may further widen global inequities in mNGS capacity. Over the past two decades, several important infectious disease outbreaks have highlighted the importance of establishing widespread sequencing capacity to support rapid disease detection and containment at the source. Using lessons learned from the COVID-19 pandemic, LMICs can leverage current momentum to design and build sustainable mNGS programs, which would form part of a global surveillance network crucial to the elimination of infectious diseases.

## Introduction

Pathogen metagenomics in public health and outbreak response has grown in popularity since the term “precision public health,” describing the use of individualized data to tailor public health interventions for target populations, was coined in 2015 (1). Metagenomic next-generation sequencing ([m]NGS) is the process of sequencing all genetic material in a biological sample including commensals and environmental contaminants, as well as disease-causing pathogens (2). However, despite major advances in NGS techniques, significant decreases in the costs associated with sequencing, and availability of more rugged sequencers, necessary skills to prepare sequencing libraries and sufficient bioinformatics capabilities for timely analysis are still a challenge in low- and middle-income countries (LMICs).

Yet, LMICs are precisely where mNGS surveillance can best detect and contain novel and emerging pathogens. Based on increased flora and fauna biodiversity and population mass, heatmaps of risk consistently show the areas at highest risk for outbreaks in Southeast Asia, India, and southern China (3). Recent examples add urgency to the task of expanding mNGS capacity in these areas: implementation of NGS in field settings proved crucial to the containment of the recent Ebola virus epidemic in West Africa as well as development of countermeasures (4, 5); metagenomics also played a significant role in the initial identification of SARS-CoV-2 (6) and potential zoonotic reservoirs (7, 8) and continues to guide outbreak response.

In this scoping review, we set out to describe the current state of mNGS programs for pathogen detection in LMICs and propose a roadmap for their future expansion (Box 1). We highlight the rationale for establishing mNGS infrastructure in LMICs, examples of its successful application in resource scarce settings (9, 10), the anticipated and surprising obstacles and attitudes to mNGS implementation, the critical need for accessible bioinformatics pipelines and expertise (11), and the impact of pre- and post-COVID experiences on mNGS feasibility in low- and middle-income countries. We conclude with mNGS lessons learned for a variety of public health aims and the ultimate goal of providing a foundation for both disease elimination and outbreak readiness strategies.

**Box 1:** Summary of published reviews and objectives and added value of this review.

**Evidence before this study**	**Study objectives**
To identify previous reviews of metagenomic next-generation sequencing (mNGS) technologies in low- and middle-income countries (LMICs), we searched the evidence available in the Medline database and consulted with content experts. Published works have previously examined the implementation of sequencing targeting specific pathogens/pathogen groups (e.g., HIV and foodborne infectious illness), and non-communicable disease. To our knowledge, there have been no comprehensive reviews of NGS applications across pathogen and infectious disease groups in LMICs, and specifically no reviews of metagenomic techniques in this context within published literature.	In this study we aim to provide an overview of the breadth of existing mNGS applications for pathogen detection and containment in LMICs, identify obstacles in the expansion of mNGS capacity in LMICs, and propose solutions to overcome these hurdles. **Added value of this study** This is the first study to review mNGS applications for pathogen detection in LMICs. This summary of contemporary data in the wake of SARS-CoV-2 provides a unique and timely perspective that recognizes opportunities for technological expansion in the post-pandemic future.

## Successful applications of mNGS in disease detection and containment

mNGS has gained popularity over the past decade as a novel diagnostic and epidemiologic tool within well-resourced settings, where applications of mNGS include outbreak investigation and antimicrobial resistance surveillance. Several groups have demonstrated that mNGS can also be successfully implemented in low-resource settings, with important downstream implications for disease recognition, control, and eradication. A comprehensive review of the literature was performed to identify reports of in-country mNGS studies conducted in LMICs (Supplementary Data); select cases are highlighted here.

### Pathogen detection in clinical settings

mNGS is filling an industry niche for the evaluation of fever of unknown origin (FUO) where other advanced microbiologic, serologic, and molecular methods failed to yield a diagnosis. The role for pan-pathogen diagnostics is best described in immunocompromised patients who are susceptible to a wide variety of opportunistic pathogens and often have surprising or non-specific disease manifestations (12). mNGS may obviate the need for multiple laboratory analyses, making it attractive for testing of small-volume clinical samples from difficult-to-sample compartments such as the central nervous system and for detection of co-infection (13). Outside of academia, commercial for-profit mNGS laboratories perform FUO analyses; for example, one company identified pathogens causing febrile neutropenia in Brazilians leading to initiation of targeted therapy in patients (14). However, prohibitive shipping costs to overseas labs and long turnaround times preclude routine clinical use of available commercial tests by LMICs. The following case studies demonstrate the feasibility and benefits of near-patient mNGS in under-resourced settings.

Pan-pathogen testing can bypass limited availability of diagnostics in low-resource settings, where rudimentary testing toolsets may miss a variety of occult or emerging pathogens. In cases of severe acute respiratory illness and meningoencephalitis in Malaysia, Vietnam, and Bangladesh, mNGS succeeded where available diagnostics had failed to identify common viral pathogens (10, 15, 16). Application of mNGS is especially attractive in HIV-prevalent countries for identifying difficult-to-detect opportunistic pathogens. In Uganda, mNGS of cerebrospinal fluid from 368 HIV-infected individuals identified 81 pathogens including unexpected cases of *Nocardia brasiliensis* and Wesselsbron virus (17). The same study also demonstrated the versatility of mNGS for detecting important co-infections (e.g., tuberculosis and toxoplasmosis).

Many LMICs are endemic for viruses causing seasonal or sporadic disease outbreaks. Disease diagnosis, particularly during large epidemics, is often made by clinicosyndromic case identification without laboratory confirmation. Differentiating etiologic agents based on clinical presentations can prove challenging given similarities in disease manifestation; even when serologic assays are available, these may be confounded by cross-reactivity. mNGS provides a method to disentangle infectious mimickers. In Cambodia, mNGS identified chikungunya in 10 patients initially diagnosed with dengue based on symptoms alone. This allowed the Cambodian Ministry of Health to rapidly incorporate chikungunya polymerase chain reaction (PCR) into national surveillance efforts and resulted in subsequent characterization of an outbreak that reached most of Cambodia's provinces within the next 2 months (9). In Brazil, mNGS evaluation of samples from patients with presumed dengue uncovered a concurrent cryptic outbreak of parvovirus B19 (18). During a 2018 presumed Lassa virus outbreak in Nigeria, mNGS identified multiple cases of yellow fever, leading to an outbreak declaration within 4 days of receiving samples (19).

The unique infectious disease landscapes of LMICs are an exciting venue to explore the expansive range of mNGS technologies. Many infectious agents commonplace in LMICs are rarely seen in most high-income countries (HICs); the lack of commercial interest in these diseases, commonly labeled “neglected tropical diseases,” has contributed to a shortage of reliable commercial diagnostics. Vector-borne diseases are a prime example. In 2020, six febrile forest workers in Cambodia were found to have parasitemia on blood microscopy, thought to be morphologically consistent with *Plasmodium malariae*. Rapid diagnostic tests targeting common malarial antigens were negative in all six individuals. Subsequent mNGS analysis reattributed the infections to *Plasmodium knowlesi*, an agent of zoonotic malaria. These findings led to incorporation of *P. knowlesi* PCR into national diagnostic algorithms (20). Scrub typhus and murine typhus, caused by obligate intracellular bacteria of the *Orientia* and *Rickettsia* genera, respectively, are traditionally diagnosed *via* fallible serologic tests. While PCR-based methods have been developed, these are not routinely available, and sensitivity varies depending on bacterial load and disease time course. mNGS may hold promising applications for identification of these pathogens (9). Lastly, mNGS can identify known pathogens manifesting with novel clinical presentations. In Bangladesh, mNGS of cerebrospinal fluid from several children presenting with idiopathic meningitis ultimately identified neuroinvasive chikungunya virus, leading to recognition of a meningitis outbreak that informed diagnostic frameworks at local hospitals (10).

### Outbreak investigation

Over the past two decades there have been several significant outbreaks of hemorrhagic fever viruses in sub-Saharan Africa, with scattered exported cases and fears of pandemic potential triggering global responses (4, 5). In these settings, in-country sequencing and analysis provided near real-time data that was rapidly translated into public health interventions. In 2008, use of mNGS enabled identification and characterization of a novel arenavirus, Lujo virus, as the etiologic agent in an outbreak of unexplained hemorrhagic fever in South Africa (21). During the 2013–2016 Ebola outbreak in West Africa, rapid expansion of in-country sequencing capabilities bypassed the lengthy turnaround times and prohibitive costs of sample exportation without loss of fidelity (5). Equipped with large benchtop sequencers in dedicated genomics centers and smaller, portable sequencers in the field, countries including Liberia and Sierra Leone used mNGS to trace viral origin and transmission patterns during the outbreak (4). Nigeria deployed mNGS during the 2018 Lassa fever season to investigate reasons for the unprecedented case surge; findings of a diverse viral population rather than a novel or divergent genotype suggested multiple distinct cross-species transmission events and highlighted the need for rodent reservoir control and improved sanitation (22). In addition to the public health benefits, these applications of mNGS in sub-Saharan Africa resulted in a wealth of sequence data critical to understanding hemorrhagic fever virus origins, diversity, and distribution (23).

### Biosurveillance

Many LMICs are hotspots for emerging infectious diseases due to their biodiversity, multiple geographic and cultural interfaces between human and zoonotic reservoirs, and an abundance of arthropod and mammalian vectors (24). mNGS provides a means for population-level surveillance of the disease landscape, allowing for detection of emerging pathogens even prior to outbreaks. In Uganda, researchers used mNGS to evaluate samples from febrile children and identified a novel orthobunyavirus and two novel human rhinovirus C (HRV-C) species (25). During the study period, a lethal HRV-C outbreak occurred in a population of chimpanzees in western Uganda. Availability of comprehensive genomic data enabled comparison of assembled HRV-C genomes; little homology between human and chimpanzee sequences implied that the events were unrelated and required no immediate public health action.

Application of mNGS to study animal and environmental viromes and microbiomes holds promise for pathogen discovery upstream of spill-over into human populations. In Uganda, viral metagenomics applied to screen domestic pigs for African swine fever virus detected Ndumu virus, an alphavirus known to infect mosquitoes and potentially transmissible to humans through this vector (26). In 2015, as part of routine testing for potential pathogens in animals within a non-human primate quarantine facility in the Philippines, mNGS helped detect the re-emergence of Reston virus in 10 macaques (27). While Reston virus has yet to demonstrate virulence in humans, the virus closely resembles human ebolaviruses and infection is highly morbid in non-human primates; it is listed as a biosafety level-4 organism in recognition of the importance of cautious handling and enhanced surveillance to detect potential mutations conferring increased pathogenicity (27).

Studying arthropod vectors can also provide surprising insights on pathogenesis and identify targets for intervention along disease transmission chains. During the 2015–2016 Zika outbreak in Barbados, mNGS of local *Aedes* mosquitoes produced two ZIKV genomes, one with a novel mutation. Mosquito sequencing identified the primary vector species in this outbreak and also suggested that predominance of a viral strain in the mosquito population could predict the strain's role in current or upcoming outbreaks (28). In Inner Mongolia, a survey of ticks identified *Dermacenter nutalli* as a potential vector of emerging spotted fever group *Candidatus Rickettsia tarasevichiae* (29). mNGS programs in LMICs are a crucial component to creating a global network of biosurveillance. With continued investment in capacity building, rapid recognition and containment of emerging threats could potentially prevent the next devastating pandemic.

### Antimicrobial resistance

Antimicrobial resistance (AMR) is a leading global health issue. In 2019, AMR accounted for over 4 million deaths, surpassing HIV and malaria in global causes of death (30). The burden of mortality is disproportionately borne by LMICs, where AMR prevalence is high and patients are often unable to afford second-line treatment. Inappropriate antibiotic use in humans and livestock, poor sanitation, substandard hospital infection control practices, and circulation of counterfeit antibiotics have been proposed as drivers of higher AMR rates in LMICs (31). Global travel and commerce mean that this problem is not restricted to LMICs. Over the past decade, several important genes conferring multi-drug resistance have arisen in LMICs, with subsequent global spread (32, 33).

Antibiotic susceptibility testing has traditionally involved time-consuming bacterial culture and identification, followed by *in vitro* exposure of isolates to antibiotics. While molecular PCR-based methods to identify resistance markers are growing in popularity, these still require targeted testing using prespecified panels for known or common pathogens. mNGS provides a rapid, comprehensive assessment in situations where unknown and/or multiple isolates and resistance mechanisms may coexist. The location of AMR genes (e.g., on highly transmissible mobile genetic elements) can be quickly identified and used to direct infection control efforts. Where increased sensitivity is needed, target enrichment can be performed within mNGS assays to detect known AMR genes including in polymicrobial samples (34).

Although still in its infancy, the field of microbiomics is growing rapidly as new applications are recognized. Studying the resistance patterns (i.e., the “resistome”) of bacteria in humans and the environment can be used to gauge the presence of clinically relevant AMR and anticipate potential spread and acquisition of transmissible AMR genes. Multinational studies have demonstrated the vast diversity of human microbiomes and resistomes across the globe with clustering in high-income vs. low-income countries and urban vs. rural communities (35). The H3Africa Initiative supported a recent study representing the largest metagenomic dataset of African adults published to date, in which stool samples from two South African cohorts dominated by different lifestyles and cultural practices (traditional vs. transitional, “westernized”) were found to have distinct AMR profiles (36). The same study found previously undescribed taxa in participant gut microbiomes, demonstrating the importance of sampling in LMICs and revealing limitations of reference collections that are dominated by contributions from HICs (36). Popularized during the SARS-CoV-2 pandemic, wastewater surveillance *via* mNGS can also be used to gauge AMR incidence and prevalence in populations to inform containment efforts, without the need for laborious individual testing (37). In Latin America, a study comparing human and wastewater samples from El Salvador and Peru revealed large networks of AMR transmission among microbial communities of humans, co-located animals, and their shared environments (38).

## The COVID-19 experience and its impact on mNGS in LMICs

The advent of COVID-19 propelled mNGS into the limelight as a means to rapidly characterize emerging pathogens, including in low-resource settings (39). SARS-CoV-2 was identified as the causative agent of COVID-19 using an agnostic whole-genome sequencing approach early in the pandemic (6). Availability of genomic data enabled its rapid classification as a sarbecovirus with genomic and clinical similarities to SARS-CoV-1 and facilitated phylodynamic analysis for the identification of potential viral origins, zoonotic reservoirs, and transmission chains (39–41). Unfortunately, by the time this information was available, the virus was widespread.

Following initial metagenomic characterization of the virus, sequence data was used to design targeted molecular diagnostics and therapeutics (42). Early knowledge of the viral genome accelerated the development of COVID-19 vaccines, likely averting countless deaths as mRNA vaccines have been the single most beneficial intervention for SARS-CoV-2 (43). A welcome byproduct of mNGS use early in the pandemic was the identification of co-infections when the clinical spectrum of COVID-19 was not yet fully understood (44). Even as the pandemic continues, genomic surveillance is being applied to identify novel variants of concern and anticipate successive waves. Viral mutation and escape from existing molecular tests and therapeutics emphasize the importance of continued availability of unbiased whole-genome sequencing to quickly detect important mutations and design countering targeted interventions (45, 46).

Since the onset of the COVID-19 pandemic, widespread sequencing of SARS-CoV-2 has led to a “genome explosion” with an unprecedented amount of sequence generation and sharing on public databases (47). LMICs with pre-existing mNGS programs including South Africa, Madagascar, Brazil, India, Bangladesh, Cambodia and Nepal were able to perform rapid in-country sequencing of the virus (39, 48, 49) and implement centralized genomic surveillance leading to early identification of several important variants of concern including B.1531 (Beta), P.1 (Gamma), B.1.617.2 (Delta), and B.1.1.529 (Omicron) (50–52). However, as of July 2022, only 3.0% of the over 11 million COVID-19 sequences uploaded to sequence database GISAID were shared by LMICs (Table 1 and Supplementary Table) (53). Recognizing a geographic disparity in global sequencing contributors with under-representation from LMICs, the World Health Organization (WHO) and Rockefeller Foundation co-initiated the Access to COVID-19 Tools Accelerator Genomic Surveillance Working Group with a goal of augmenting sequencing capacity across the globe (54). On a regional scale, multilateral organizations including Africa Centers for Disease Control and Prevention (Africa CDC), Pan American Health Organization (PAHO), and Association of Southeast Asian Nations (ASEAN) partnered with several public, private, and non-profit organizations to create regional surveillance networks and support sequencing in member nations. The global solidarity and pressures generated by the COVID-19 pandemic promise to usher in a new era with sequencing commonplace and integrated into national diagnostic and surveillance frameworks in HICs and LMICs alike. Beyond the pandemic, the challenge will be to maintain interest in these technologies to ensure their sustainability.

**Table 1:** Number of SARS-CoV-2 sequences shared to GISAID as of July 26th, 2022 by country/territory and economic status, with top 15 countries in each category listed [Data source: https://www.gisaid.org/submission-tracker-global/ (accessed July 26, 2022)].

**Country/territory**		**Number of sequences**
**High-income economies**		
	United States of America	3,734,418
	United Kingdom	2,764,684
	Germany	715,173
	Denmark	542,561
	Canada	404,512
	France	398,007
	Japan	318,929
	Sweden	198,749
	Austria	148,351
	Spain	146,814
	Switzerland	145,417
	Belgium	138,241
	Italy	136,867
	Australia	133,053
	Netherlands	130,113
**Low- and lower-middle-income economies**		
	India	212,558
	Indonesia	29,609
	Philippines	21,125
	Kenya	10,983
	Bangladesh	7,197
	Nigeria	6,823
	Vietnam	5,586
	Papua New Guinea	4,382
	Senegal	3,909
	Ghana	3,891
	Pakistan	3,540
	Sri Lanka	3,451
	Cambodia	3,253
	Nepal	3,193
	Iran	2,392

*A full version including all countries is provided in (Supplementary Table). Country/territory income classification were determined using World Bank definitions: https://datahelpdesk.worldbank.org/knowledgebase/articles/906519-world-bank-country-and-lending-groups*.

## Obstacles

Despite demonstration of mNGS feasibility with numerous impactful successes, various obstacles unique to these settings thwart the uptake of mNGS in LMICs and widen the equity gap between low- and high-resource nations.

### Access to a pool of skilled personnel

National health research capabilities (including clinical trials, funding attraction, publications, and doctorate training) show disparities between HICs and LMICs (55). The development of advanced research hubs requires access to a pool of skilled scientists; in LMICs, this represents a major barrier to expansion of research capabilities (56). For example, six sub-Saharan African countries, all countries with high health research capabilities relative to other countries in the region (55), had substantially fewer researchers per million than the global average (3.5–39.8%) (57).

More specifically, paucity of researchers trained in bioinformatics is a major barrier to expanding mNGS capabilities in LMIC institutions. Conversely, availability of bioinformaticians in HICs may further widen disparities (58). Undergraduate and postgraduate bioinformatic programs are not available in all educational centers and are not equally distributed even within continents (59). Countries lacking structured pedagogical systems may struggle to maintain the skilled workforce needed to run advanced biotechnology programs. Multiple initiatives have tried to fill this gap by providing training in bioinformatics for biologists and related scientists in LMICs. However, workshops and courses require significant resources and collaboration to organize and deliver (60).

Competing demands further hinder the ability of trained scientists to use these skills to perform research. Scientists in universities in Kenya and South Africa had high teaching and supervision workloads compared to European and North American counterparts (61). In addition, while retention has been overall good in the U.S. (70% of CDC/Association of Public Health Laboratories recruits are still working in public health) (2), retention rates of biologists trained in bioinformatics in LMICs are unknown but may be challenged by scarcity of well-developed post-doctoral programs, poor working conditions and wages, and—in extreme conditions—war.

### Access to platforms and reagents

Apart from cost, which will be discussed later, access to platforms is a combination of the ease of use of the platform and availability of (i) the sequencing instrument plus the flow cells and reagent kits to operate it, (ii) routine and emergency service/maintenance of instrument(s), and (iii) reagents and protocols required to prepare samples for sequencing. Illumina and Oxford Nanopore Technologies (ONT) are currently the most prevalent platforms for mNGS in LMICs. While there are several other mNGS platforms on the market, due to the space constraints of this manuscript, only these two platforms will be discussed in detail.

Illumina provides a wide gamut of short-read sequencers that yield 1.2 Gb to several Tb of data, with the iSeq being the smallest sequencer (<1 ft^2^). The disposable reagent cartridge and flow cell of this instrument include all fluidics and optics required for sequencing, making the iSeq the most maintenance-friendly instrument of Illumina's lineup and hence attractive for regions where access to service engineers and support is limited. The minION, the smallest sequencer from ONT, can generate up to 50 Gb of data, albeit with a lower raw read accuracy than Illumina sequencers (62). The portability and lower cost of the minION make it an attractive platform, especially for settings with limited resources and infrastructure. The larger ONT instruments still have a limited footprint compared to non-iSeq Illumina instruments, and their maintenance requirements remain minimal due to the use of disposable all-in-one flow cells. Illumina instruments generally have built-in computing power for initial analyses such as base-calling and demultiplexing, while most ONT instruments require extra data processing units and steps after data collection to generate fastq data files containing interpretable sequence data.

Acquiring a sequencer is one obstacle, ensuring a predictable and timely inflow of all reagents needed to run that sequencer is another. Most HICs have direct access to instruments, reagents, and service from the manufacturers themselves through national or regional offices, but access in LMICs is most often provided through distributors. Illumina was founded in 1998 and has several commercial offices that provide the U.S. and the Europe-Middle East-Africa region (all located in Europe) with direct customer service, ensuring short order fulfillment cycle times. However, South America, Southeast Asia, and East Asia each have just a single commercial Illumina office, and most LMICs are primarily serviced through local third-party distributors with variable quality in customer service and robustness of supply chains. ONT was founded in 2005 and has fewer regional offices but also employs several distributors, some that service several countries at once in Europe and sub-Saharan Africa. Many LMICs do not have an allocated local distributor for any sequencing products in place despite having established sequencing capacity. Conversely, local distributors servicing LMICs do not have the broad customer base that HICs' distributors may have; as they are required to take on significant financial risks, this often leads to higher prices, low inventories, and extended order fulfillment cycle times. In addition, most reagents require shipping at <20°C which can be challenging in the context of higher ambient temperatures and/or extended delivery times. Finally, access to instrument maintenance by knowledgeable service engineers is especially challenging in underserved LMICs. This not only results in extended instrument downtime in LMICs vs. HICs, but also limits local sequencing groups in their efforts to expand their throughput with higher-end instruments beyond the low-maintenance, easy-to-use iSeq and minION type of sequencers.

Preparing samples for sequencing, a process often referred to as library prep(-aration), also requires specific reagents. Procuring these reagents from the same sequencing company (or distributor) that also provides the required flow cells and sequencing cartridges is efficient from a supply chain perspective. On the other hand, procuring compatible kits from other life science companies may be cheaper and allow for more customized orders. However, having to order compatible reagents from yet another distributor adds complexity to already existing supply chain issues in LMICs. Of note, not all sequencing workflows can be customized to the same extent—e.g., alternative barcoding kits allowing for multiplexing are available for Illumina but not ONT workflows due to the requirement of specific motor proteins on ONT adapters. Flexibility in library prep workflows is an important tool to cope with slow or unreliable supply chains and reduce interrupted access. This includes different types of kits for library prep, different approaches to library prep QC, and miniaturizing reactions as much as possible to maximize the number of reactions that can be performed with a single kit. While many such protocols exist, they are often hard to find.

### Access to analytical pipelines

mNGS sequencing produces gigabases of data, making analysis and data storage computationally intensive and expensive. There has been no standardization of available mNGS pipelines, resulting in a myriad of tools and databases specializing in topics from microbial ecology of environmental samples (e.g., MG-RAST) (63) to viral pathogen detection in clinical samples. Unfortunately, most of these tools require bioinformatics expertise and strong command-line skills, making the barrier to entry high and often insurmountable in areas with limited resources.

There are several graphical user interface (GUI) -based user-friendly options available that circumvent requirements such as coding fluency. Tools such as Genome Detective (64). One Codex (65), and DNASTAR's Lasergene (66) provide push-button analysis of raw data to taxonomic identification and abundances; however, they also require paid subscriptions which may preclude their use in labs that are already stretched for resources. Free options such as CZ ID (11), Nephele (67), and Taxonomer (68) are available, with Taxonomer offering a paid subscription to view visualizations such as read coverage of an accession.

One of the main difficulties of mNGS analysis is the ability to identify contamination and differentiate between true and spurious hits. Tools such as CZ ID offer visualizations and filters to help users remove spurious hits from their analysis and gain confidence in true hits. Access to figures such as heatmaps, background models (69), and coverage visualizations are key to identifying important pathogens.

Although visualizations are essential in identifying and confirming pathogens, the results of the pipeline are only as useful as the underlying reference database. Choosing a tool that compares sequences against a comprehensive database such as the nucleotide and protein databases hosted by NCBI (for mNGS analysis) increases detection sensitivity. However, pathogen landscapes are region-specific and pathogens from LMICs may be underrepresented in public databases since NGS capacity is often lacking. For example, *Burkholderia pseudomallei*, a bacterial pathogen that causes melioidosis, is common in Southeast Asian LMICs but severely underrepresented in AMR databases (70).

### Access to internet

The internet is instrumental in accessing training opportunities and a network of collaborators, and it is also at the center of mNGS data sharing and analysis and tool development (71). Data transfer is necessary to share knowledge, enable cross-institutional collaborations, and alleviate the need of bioinformatic expertise if cloud-based analytical tools are available. Nevertheless, internet access remains a major barrier in LMICs (72). In 2021, 2.9 billion people did not have internet access; of these, 96% lived in LMICs (73). Broadband speed is lowest in LMICs, and while average speeds are increasing, so are data sharing needs (74). E-infrastructure can be costly and prohibitive in some settings, and even in places where internet can be secured, transfer times can be excessive (75). In addition to overall low speeds, internet connectivity can be unreliable and subject to government control; during times of social unrest and security concerns, governments resort to shutting down most or all internet access (76). Furthermore, power supply can be erratic, making it more challenging to perform sequencing, upload data, and perform analysis (72). As a result, LMIC institutions look for computational solutions that can operate independently of internet access, though this is complicated by the substantial lack of computer infrastructure and skilled personnel to manage large amounts of data (e.g., as of 2018, only a few African countries had high-performance computing capabilities in select institutions) (77).

### Financial cost

The cost of sequencing has dropped precipitously over the past few decades. In the early 1990's, the Human Genome Project, designed to sequence the entire *Homo sapiens* genome, cost US$2.7 billion and took over 13 years to complete (78). By 2010, the complete human genome could be sequenced for US$10,000, and in 2022 the cost has dropped to a mere US$600, equivalent to about US$3 per gigabyte of sequence data (79). Excluding startup and ancillary expenditures, “homebrew” mNGS of a single sample run as part of a multiplexed reaction with high-throughput processing costs ~US$10–100 depending on platform, sample type (i.e., nucleic acid density, RNA vs. DNA, read length), sample processing (e.g., removal of non-target reads, enrichment), and sequencing breadth and depth required, among other factors (80, 81). Commercial laboratories in the U.S. charge in the range of US$2000–3000 for mNGS of a clinical sample (82). In contrast, pathogen-directed rapid diagnostic tests can cost as little as US$1, but even these are often out of reach for many people living under the poverty margin in LMICs. Although continued refinements in sequencing technologies will further reduce associated costs, individual affordability may continue to be a barrier in pay-for-use healthcare systems.

At the population level, startup and maintenance costs of mNGS programs are often prohibitive for many LMICs, and more immediate public health needs may relegate such longer-term investments to the backburner. Disparities in program costs between LMICs and HICs add to a growing inequity in mNGS capabilities based on country GDP. In LMICs, activation costs for mNGS programs must cover infrastructure development, workforce creation, and build-up of computing capacity in addition to standard costs of laboratory equipment and consumables (83); where programs have succeeded, it has been mostly due to an initial injection of capital from philanthropic and/or academic groups (84, 85). Maintenance of sequencing programs may also be more costly in LMICs vs. their high-income counterparts due to outsourcing of data storage, bioinformatics analysis, and technical assistance, frequent instrument malfunction due to suboptimal storage conditions, and high tariffs on imported reagents and equipment (86). While mNGS has incredible potential for advancing public health and associated economic benefits in LMICs, the immediate costs may be perceived as disproportionate to the benefits that are often reaped much later.

### Time cost

Time-sensitive situations with rapidly evolving communicable disease outbreaks demand prompt sequencing turnaround. Delays at several stages can hamper sequencing efforts and interrupt responses to clinical and public health emergencies. Transporting samples across long distances to centralized sequencing hubs may require substantial time in regions with poor transport networks and infrastructure and amplify inherent hazards of sample mishandling (87). Reagents needed to collect, pack, and process samples often have high procurement lead times and are subject to frequent stockouts as a result of weak regional distribution and poor local inventory management (88). Civil unrest may further disrupt transportation routes, compounding shortages of consumables. Bureaucratic hurdles add to time cost despite multinational efforts to facilitate cross-border resource sharing. The Nagoya protocol was adopted in 2010 as a multinational treaty to regulate cross-border equitable access to genetic resources and benefits arising from their use. To date, the protocol has 133 signatories with robust representation from LMICs (89). Although well-intentioned, the Nagoya protocol has been criticized for its unclear definition of genetic resources, reliance on bilateral negotiations and convoluted documentation, and lackluster efforts by signatories to incorporate protocol conditions into their national legal systems (90); detractors may also tout its utilitarian view of public health promotion at the price of informed patient consent. Similar networks exist at regional levels [e.g., the Asian Consortium for the Conservation and Sustainable Use of Microbial Resources (ACM)] and may offer simplified processes albeit on a smaller, local scale (90).

### Access to data

Despite the growing number of studies and applications of mNGS in LMIC, pre-COVID-19 publications of metagenomic sequencing studies and data were dominated by HICs (91). Recent calls for democratization of data generated in LMICs, but ultimately used for analyses, applications, and product development in HICs, have resulted in multilateral initiatives and policy directives such as the previously discussed Nagoya Protocol (90), the Global Virome Project (92), and others. Aside from the economic constraints of purchasing supplies and equipment in LMICs, the intangible or intellectual drivers behind the discrepancy in published studies from LMICs are variable. At the individual level, LMIC investigators may be rightfully reluctant to share data whose exclusivity results in higher-impact publications, and thus career progression and access to grant funds (93). Countries may also be wary of unrestricted international access to potentially stigmatizing local data; a notable example was the widespread implementation of strict travel restrictions for South Africans after the country was first to report the SARS-CoV-2 Omicron variant in November 2021 (94). At a systems level, there are two major barriers to democratizing access to data: (1) restricted open data repositories and (2) fee-for-service publication and access.

Open data repositories, such as GISAID, were popularized during influenza and SARS-CoV-2 pandemics because they rightly require that credit be given to the groups who initially performed both the wet and dry lab analyses resulting in the pathogen genome (95, 96). This is in contrast to GenBank, a more “carte blanche” open access data repository hosted by the U.S. National Institutes of Health. However, the well-intended built-in mechanisms at GISAID result in data access constraints, particularly for those in LMICs who may lack institutional email addresses and thus be denied access to the genomic data. Additionally, some scientists have lost access after failing to appropriately credit the organization, not using approved names, or mixing data from other repositories.

The second major challenge to access revolves around not the raw sequence data, but the curated approaches and conclusions resulting from said data that appear in peer-reviewed publications. The COVID-19 pandemic democratized access because all major journals made COVID-19-related material freely available, and global use of preprint servers for submitted manuscripts soared (97, 98). Prior to these trends, LMIC investigators relied upon dedicated open access journals, like the PLOS series, or individual articles, an optional additional expense for an author to publish their work to be accessible to all. The website ResearchGate mitigated some of these access issues in LMICs by allowing authors to upload these PDFs themselves, but the website is involved in constant legal battles over copyright infringement with academic journals for its practices (99). In sum, if sequence data and robust sequencing platforms are to play a role in disease elimination, then equally robust data sharing policies and platforms must be in place.

## Sustainability

**Figure 1:**
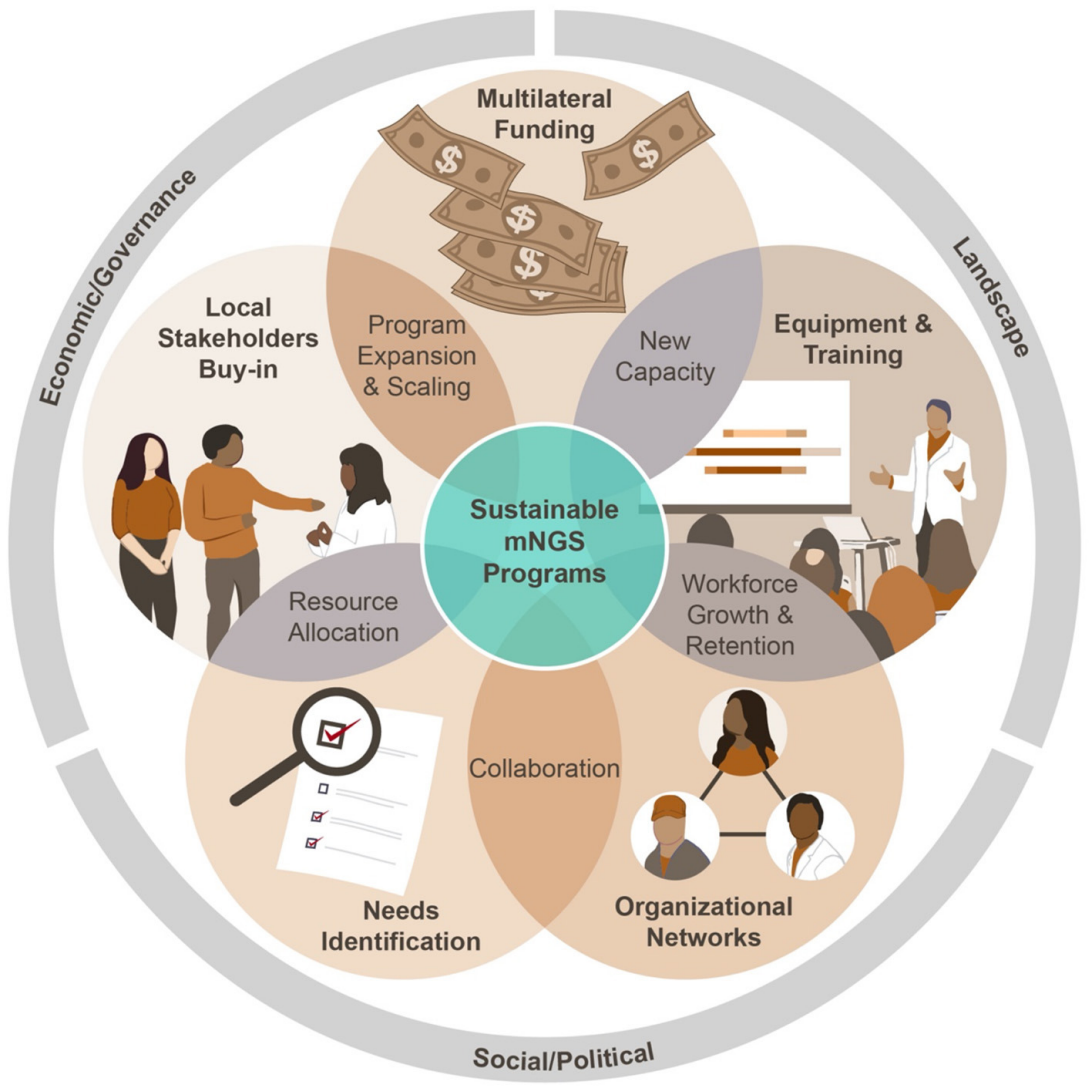
Key components of a sustainable mNGS program.

Beginning with the genomic revolution in the early aughts and in the wake of devastating outbreaks including SARS-CoV-2, Zika, and Ebola viruses, global prioritization of genomics as part of surveillance is increasing, particularly for emerging infectious diseases. The One Health approach, which emphasizes integration of public, private, and governmental sectors to implement public health initiatives, provides a model on which to design successful mNGS programs with the goal of constructing a global surveillance network. Here, we identify and summarize several key components to ensuring program sustainability (Figure 1).

### Building a workforce

**Figure 2:**
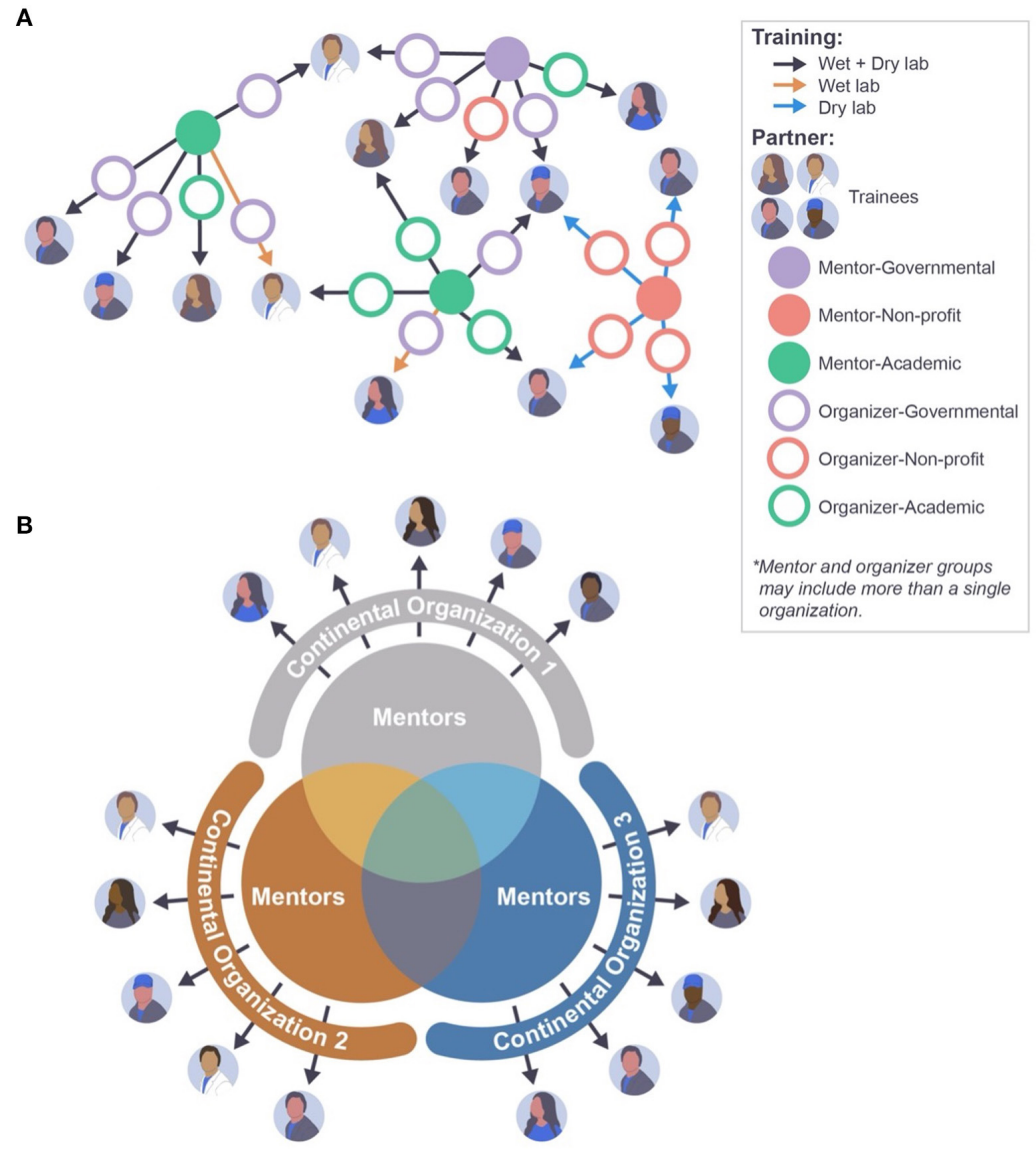
Approaches to mNGS training and capacity building. **(A)** Current training landscape lacks central coordination, causing inefficiencies such as varying training levels and duplications of effort. **(B)** Continental organizations can coordinate the training efforts of a pool of mentors, allowing for a more systematic and standardized approach to NGS training and capacity building.

Current training infrastructure in LMICs receives input from a number of groups including academic, public, and NGOs, with significant redundancy and lack of standardization and centralized quality control (100). These efforts are frequently siloed, leaving individual investigators to piece together a variety of workshops and coursework to formulate an adequate knowledge base, often without supplementation by comprehensive hands-on training and mentorship (Figure 2A). Though effective in the short term, these methods can suffer from a lack of continuity due to the transiency of external expert personnel, attrition of locally trained staff, and a lack of comprehensive technical support while putting newly gained knowledge into practice. Ultimately, these factors often work to dismantle a program, leaving new technology to languish and remain underutilized.

Investment in skillset acquisition and development of personnel through parallel, organized efforts that are coordinated to teach preset curricula are imperative for success (Figure 2B). Trainer and workshop alignment and partnership within consortium programs can help promote better evaluation and monitoring of the overall curriculum (101, 102). Online learning, including frameworks established during COVID-19 pandemic lockdowns (103), can be leveraged to provide flexible access to standardized, vetted training programs across borders, with the added benefit of fostering professional relationships and multilateral growth. In this defining era of virtual education, such investments in human capital can be uniquely profitable, and their effects long-term.

### Removing financial barriers

Cost is an overwhelming hurdle to the broad implementation of mNGS in LMICs. Many LMICs rely on donations and financial support from global partners to support local disease control efforts, with funds mostly directed toward high-visibility diseases including malaria, HIV, and tuberculosis. During the pandemic, existing disease control programs were adapted in many instances to address COVID-19-specific needs in LMICs (104). Next steps could mirror the U.S. President's Emergency Plan For AIDS Relief (PEPFAR) and Global Ebola Response coordinated funding approaches: COVID-specific influx of money can be appropriated to bolster sequencing capacity that will serve a country or region's infrastructure well after the pandemic has ended (105). Additionally, funding dedicated to COVID-19 pandemic response can be put toward establishing comprehensive bioinformatics training coordinated across public- and private-sector sequencing agents, equipping LMICs with a new generation of skilled personnel. Another step toward sustainability could involve establishing a tiered-funding sector-wide approach (SWAP) similar to that for HIV in the wake of PEPFAR implementation, which allows for a country to fund what is needed (reagents, staff, equipment) without limitations on how funds can be used (106). As country GDP increases, sequencing financial support from the regional entity is appropriately decreased.

Beyond procuring financial support, reducing inherent costs and eliminating inequitable trade are vital to making advanced technologies like mNGS feasible in LMICs. The price tag on sequencing has dropped precipitously over the past decade with the decline largely attributable to intrinsic market forces and technological advances. Continued support from stakeholders in industry, academia, government, and non-governmental organizations can further increase affordability of consumables, equipment, analytical software, and skilled labor (78). Multilateral geopolitical organizations play an important role: entities like WHO, Africa CDC, ASEAN, and PAHO can work with suppliers to support R&D efforts and improve affordability of consumables as was done during the COVID-19 pandemic to equitize access to diagnostic testing (54). These organizations can also encourage inter-governmental trade agreements focusing on reducing tariffs on items for public health-related sequencing initiatives, while coordinating resource distribution to minimize inventory stockpiling and stockouts. Such effective oversight would streamline commercial and diplomatic negotiations, an undertaking further facilitated by adapting and improving existing frameworks such as the Nagoya protocol.

### Defining public health needs

Excitement surrounding mNGS rollout in healthcare and public health settings should be tempered by recognition of the role for stewardship in its application and an attempt to weigh benefits against pitfalls. Little is known about the cost-effectiveness of its use outside of pathogen detection in clinical settings (107). For LMICs recently embracing the technology, mNGS may be best applied in a targeted, controlled fashion to avoid excessive demands on finite resources. The considerable costs, both in time and money, associated with mNGS still preclude its replacement of traditional, specific tests such as PCR which are available for several endemic pathogens (i.e., dengue, malaria, HIV, and tuberculosis). Nevertheless, metagenomics is a valuable addition to the epidemiologist's arsenal. The depth of information provided by mNGS plays a critical role in pathogen identification but also in development of more rapid and portable targeted testing. The challenge is utilizing such support for not only individual pathogen detection and treatment but also surveillance and prevention on a population level. To maximize its usefulness, a balance must be achieved between periodic metagenomic sequencing (to appraise antigenic shift, for instance) and targeted, widespread testing campaigns to inform treatment and public health response (108).

Public health organizations are tasked with providing guidance on the role of mNGS in diagnostic and surveillance algorithms. The first step is to coordinate mapping studies using mNGS to characterize a country or region's unique infectious disease landscape. These can be performed by various groups (9, 25) with data collated, curated, and maintained in a central biorepository. Endemic public health priorities can be derived from these data and interventions, including immunization, vector control, and resource allocation, planned accordingly. During nascent outbreaks, sequencing data should be made publicly accessible to bolster industry R&D and development of targeted diagnostics and therapeutics. Once specific tests are available, clinical diagnostic algorithms and management strategies should rely on more affordable, rapid, and readily available tests while sequencing efforts shift to focus on periodic genomic surveillance to capture pathogen evolution and escape.

### Supporting regional and international collaboration

The final key to building sustainable programs in mNGS involves the construction of collaborative regional and international networks that extend across both public- and private-sector boundaries. Overarching goals of partnerships would include: (i) providing regulatory oversight, (ii) guiding policy and implementation, (iii) facilitating equitable access to resources, and (iv) coordinating efforts through data sharing and construction of a surveillance network. A recent assessment conducted by the Africa CDC found that 71% of sequencers within the continent were concentrated in just five countries, with the vast majority residing in institutions that were not affiliated with national public health institutes (100). This assessment underscores the need to reassess how sequencing is performed beyond centralized regional hubs; comprehensive capacity mapping efforts during COVID-19 can be used as a model (109).

At a local level, stakeholders including academic institutions, commercial suppliers, and local governmental bodies must commit to developing laboratory infrastructure, opening supply chains, fast-tracking approval of time-sensitive sequencing projects, and supporting development of a skilled workforce. At a national level, regulatory agencies such as the Food and Drug Agency (FDA) and Clinical Laboratory Standards Institute (CLSI) in the U.S. should evaluate and develop standards for clinical and public health-facing mNGS technologies. Across borders, priorities lie in growing networks for distribution of information and materials. Global trade and technology platforms like Kountable and *via* Global Health have already begun to close the gap between small, local businesses and the global market, making medical and other scientific equipment accessible through technological innovation and streamlined distribution (110, 111).

Where there is inability to build mNGS capacity from scratch, establishing referral networks and facilitating sample transportation could maximize the use of regional mNGS and computing capacity (83). Consortia such as the Asia Pacific Bioinformatics Network and the Pan African Bioinformatics Network promote sharing of bioinformatics knowledge and improve access to analytical tools (112). Access to sequencing protocols, data, and publications can be democratized *via* online private- and public-sector platforms with free account options and/or enhanced access for researchers from LMICs, as with cOAlition S and WHO's Health InterNetwork Access to Research Initiative (HINARI) (113). Ethical standards should be defined early and enforced at local, national, and international levels to protect patient confidentiality and data sovereignty (114).

Once again, the role for multilateral geopolitical organizations as central coordinators of mNGS expansion should be emphasized. By facilitating equitable trade, overseeing regulation of technologies, directing funding and investments, coordinating capacity building, establishing regional referral networks, and encouraging data and knowledge sharing, the sequencing equity gap between LMICs and HICs can be narrowed to make a comprehensive global genomics surveillance network a reality.

## Conclusion

While mNGS remains a nascent technology in most LMICs, its feasibility and broad applications for the detection, control, and prevention of disease-causing pathogens have already been demonstrated in low-resource settings. Uptake of mNGS in LMICs is slower than in HICs due to the numerous identified obstacles, but these can be overcome with appropriate planning, funding, and dedication to sustainability (Box 2).

**Box 2:** Obstacles and mitigating strategies to build metagenomic next-generation sequencing capacity in LMICs.

**Obstacle**	**Mitigating strategies**
Access to a skilled workforce and supporting infrastructure	• Collaborative, coordinated education campaigns • Virtual training programs • Open-source, standardized bioinformatics pipelines • Knowledge sharing through online collaborative networks • Establish regional referral networks to harness centralized sequencing and computing capacity
Access to platforms and reagents	• Develop low-maintenance, high-output sequencing instruments with: • Disposable, all-in-one components • Adaptable reagents for cross-platform use • Reduced maintenance/servicing requirements • Fund R&D and reduce supplier costs to support priority public health programs • Improve distribution and customer support strategies • Open-source, user-validated protocols that miniaturize reactions and allow for alternative library prep workflows
Cost	• Tailor sequencing to country-specific public health needs • Establish regional distribution networks and global trade and technology platforms • Inventory management • Tiered funding • Trade agreements
Access to data	• Democratize access to scientific literature (e.g., through preprints, open access journals) • Provide equitable access to sequence repositories • Protocol sharing and regulation • Protection of appropriate credit/anonymity

While the long road to democratizing access to mNGS is daunting, there are global incentives to narrow the technological gap between LMICs and HICs. The microbial diversity found in LMICs represents a wealth of genomic data that can provide valuable scientific insights. To tap this resource, those closest to these evolving frontiers must be equipped with mNGS capacity and the resources to sustain it. Infectious outbreaks over the last decade have reinforced the importance of pathogen detection at the source to inform rapid clinical and public health interventions. LMICs should prioritize mNGS surveillance for emerging infections and antimicrobial resistance to capture potential threats before they are widespread and enable appropriate resource allocation for endemic health needs. Growing mNGS capacity in LMICs would ultimately facilitate a transition from top-down surveillance systems to a local-global model, which would optimize use of this expansive resource and benefit public health worldwide.

Consistent communication and exchange between actors from various sectors are essential to building sustainable mNGS programs. Financial contributions alone will not suffice to democratize sequencing capacity; knowledge-sharing, as well as regulation and diplomacy within and between countries, are paramount to successful mNGS implementation. Developing the infrastructure necessary to establish and sustain sequencing programs in LMICs, while complex, becomes a manageable challenge when approached with coordinated efforts across institutions. Acquiring a sequencer is no longer the greatest obstacle for laboratories in low-resource settings; instead, the feasibility of mNGS depends increasingly on the quality of available educational, commercial, and data-sharing networks. While the onus is on governmental and non-governmental leaders from countries with established metagenomics infrastructure to act, the growth of mNGS as a field will require unified, collaborative strategies as previously discussed. Such efforts on this frontier of science will bring about the next age of unprecedented discovery and learning. By deriving lessons on global solidarity from pandemic times to foster global cooperation, we can pivot from COVID-19 to expand LMIC access to mNGS and usher in a post-pandemic future.

## Author contributions

CY and AP collected and analyzed the data. CT and JM conceptualized the study. All authors were involved in drafting, reviewing, and editing the manuscript. All authors contributed to the article and approved the submitted version.

## Funding

This research was supported by the Division of Intramural Research at the National Institute of Allergy and Infectious Diseases and the Bill and Melinda Gates Foundation [grant number OPP1211806].

## Conflict of interest

The authors declare that the research was conducted in the absence of any commercial or financial relationships that could be construed as a potential conflict of interest.

## Publisher's note

All claims expressed in this article are solely those of the authors and do not necessarily represent those of their affiliated organizations, or those of the publisher, the editors and the reviewers. Any product that may be evaluated in this article, or claim that may be made by its manufacturer, is not guaranteed or endorsed by the publisher.

## References

[1] Khoury MJ Iademarco MF Riley WT. Precision public health for the era of precision medicine. Am J Prev Med. (2016) 50:398–401. 10.1016/j.amepre.2015.08.031 26547538 PMC4915347

[2] Armstrong GL MacCannell DR Taylor J Carleton HA Neuhaus EB Bradbury RS. Pathogen genomics in public health. N Engl J Med. (2019) 381:2569–80. 10.1056/NEJMsr1813907 31881145 PMC7008580

[3] Allen T Murray KA Zambrana-Torrelio C Morse SS Rondinini C Marco MD. Global hotspots and correlates of emerging zoonotic diseases. Nat Commun. (2017) 8:1124. 10.1038/s41467-017-00923-8 29066781 PMC5654761

[4] Gire SK Goba A Andersen KG Sealfon RSG Park DJ Kanneh L. Genomic surveillance elucidates Ebola virus origin and transmission during the 2014 outbreak. Science. (2014) 345:1369–72. 10.1126/science.1259657 25214632 PMC4431643

[5] Hoenen T Groseth A Rosenke K Fischer RJ Hoenen A Judson SD. Nanopore sequencing as a rapidly deployable Ebola outbreak tool. Emerg Infect Dis. (2016) 22:331–4. 10.3201/eid2202.151796 26812583 PMC4734547

[6] Wu F Zhao S Yu B Chen YM Wang W Song ZG. A new coronavirus associated with human respiratory disease in China. Nature. (2020) 579:265–9. 10.1038/s41586-020-2008-3 32015508 PMC7094943

[7] Hul V Delaune D Karlsson EA Hassanin A Tey PO Baidaliuk A. A novel SARS-CoV-2 related coronavirus in bats from Cambodia. BioRxiv. (2021) 2021:428212. 10.1101/2021.01.26.428212 34753934 PMC8578604

[8] Zhou H Ji J Chen X Bi Y Li J Wang Q. Identification of novel bat coronaviruses sheds light on the evolutionary origins of SARS-CoV-2 and related viruses. Cell. (2021) 184:4380–91.e14. 10.1016/j.cell.2021.06.008 34147139 PMC8188299

[9] Bohl JA Lay S Chea S Ahyong V Parker DM Gallagher S. Discovering disease-causing pathogens in resource-scarce Southeast Asia using a global metagenomic pathogen monitoring system. Proc Natl Acad Sci USA. (2022) 119:e2115285119. 10.1073/pnas.2115285119 35238677 PMC8931249

[10] Saha S Ramesh A Kalantar K Malaker R Hasanuzzaman M Khan LM. Unbiased metagenomic sequencing for pediatric meningitis in bangladesh reveals neuroinvasive chikungunya virus outbreak and other unrealized pathogens. mBio. (2019) 2019:19. 10.1128/mBio.02877-19 31848287 PMC6918088

[11] Kalantar KL Carvalho T de Bourcy CFA Dimitrov B Dingle G Egger R. IDseq—an open source cloud-based pipeline and analysis service for metagenomic pathogen detection and monitoring. GigaScience. (2020) 9:giaa111. 10.1093/gigascience/giaa111 33057676 PMC7566497

[12] Benamu E Gajurel K Anderson JN Lieb T Gomez CA Seng H. Plasma microbial cell-free DNA next-generation sequencing in the diagnosis and management of febrile neutropenia. Clin Infect Dis. (2021) 2021:ciab324. 10.1093/cid/ciab324 33870413 PMC9070798

[13] Wilson MR Sample HA Zorn KC Arevalo S Yu G Neuhaus J. Clinical metagenomic sequencing for diagnosis of meningitis and encephalitis. N Engl J Med. (2019) 380:2327–40. 10.1056/NEJMoa1803396 31189036 PMC6764751

[14] Garnica M Pierrotti LC Oliveira PV de Mazzi M Chebabo A. Metagenomic next-generation sequencing (mNGS) for diagnostically challenging infectious diseases in patients with acute leukemia. Braz J Infect Dis. (2021) 25:101548. 10.1016/j.bjid.2021.101548 33639095 PMC9392121

[15] Hong NTT Anh NT Mai NTH Nghia HDT Nhu LNT Thanh TT. Performance of metagenomic next-generation sequencing for the diagnosis of viral meningoencephalitis in a resource-limited setting. Open Forum Infect Dis. (2020) 7:ofaa046. 10.1093/ofid/ofaa046 32158774 PMC7051036

[16] Chong YM Chan YF Pang YK Hasan MS Jamaluddin MFH Omar SFS. Viral etiology of severe acute respiratory infections in adults in Kuala Lumpur, Malaysia. Int J Infect Dis. (2020) 101:230. 10.1016/j.ijid.2020.11.035

[17] Ramachandran PS Ramesh A Creswell FV Wapniarski A Narendra R Quinn CM. Integrating central nervous system metagenomics and host response for diagnosis of tuberculosis meningitis and its mimics. Nat Commun. (2022) 13:1675. 10.1038/s41467-022-29353-x 35354815 PMC8967864

[18] Di Paola N Mesquita FS Oliveira DBL de Villabona-Arenas CJ Zaki Pour S de Sousa-Capra C. An outbreak of human parvovirus B19 hidden by dengue fever. Clin Infect Dis. (2019) 68:810–7. 10.1093/cid/ciy630 30304533

[19] Ajogbasile FV Oguzie JU Oluniyi PE Eromon PE Uwanibe JN Mehta SB. Real-time metagenomic analysis of undiagnosed fever cases unveils a yellow fever outbreak in Edo State, Nigeria. Sci Rep. (2020) 10:3180. 10.1038/s41598-020-59880-w 32081931 PMC7035389

[20] Yek C Lay S Bohl JA Man S Chea S Lon C. Cambodian national malaria surveillance program detection of *Plasmodium knowlesi*. Am J Trop Med Hyg. (2022). 10.4269/ajtmh.22-0039 35895370 PMC9294667

[21] Briese T Paweska JT McMullan LK Hutchison SK Street C Palacios G. Genetic detection and characterization of Lujo virus, a new hemorrhagic fever–associated arenavirus from Southern Africa. PLoS Pathog. (2009) 5:e1000455. 10.1371/journal.ppat.1000455 19478873 PMC2680969

[22] Siddle KJ Eromon P Barnes KG Oguzie JU Mehta S Odia I. Genomic analysis of Lassa virus from the 2018 surge in Nigeria. N Engl J Med. (2018) 379:1745–53. 10.1056/NEJMoa1804498 30332564 PMC6181183

[23] Di Paola N Sanchez-Lockhart M Zeng X Kuhn JH Palacios G. Viral genomics in Ebola virus research. Nat Rev Microbiol. (2020) 18:365–78. 10.1038/s41579-020-0354-7 32367066 PMC7223634

[24] Keusch GT, Pappaioanou, M, Gonzalez, MC, Scott, KA, Tsai, P,. Drivers of Zoonotic Diseases. Sustaining Global Surveillance Response to Emerging Zoonotic Diseases. Washington, DC: National Academies Press (2009) Available online at: https://www.ncbi.nlm.nih.gov/books/NBK215318/ (accessed April 5, 2022). 25009943

[25] Ramesh A Nakielny S Hsu J Kyohere M Byaruhanga O de Bourcy C. Metagenomic next-generation sequencing of samples from pediatric febrile illness in Tororo, Uganda. PLoS ONE. (2019) 14:e0218318. 10.1371/journal.pone.0218318 31220115 PMC6586300

[26] Masembe C Michuki G Onyango M Rumberia C Norling M Bishop RP. Viral metagenomics demonstrates that domestic pigs are a potential reservoir for Ndumu virus. Virol J. (2012) 9:218. 10.1186/1743-422X-9-218 23006778 PMC3512490

[27] Demetria C Smith I Tan T Villarico D Simon EM Centeno R. Reemergence of Reston ebolavirus in Cynomolgus Monkeys, the Philippines, 2015. Emerg Infect Dis. (2018) 24:1285–91. 10.3201/eid2407.171234 29912712 PMC6038738

[28] Thannesberger J Rascovan N Eisenmann A Klymiuk I Zittra C Fuehrer HP. Viral metagenomics reveals the presence of novel Zika virus variants in Aedes mosquitoes from Barbados. Parasit Vectors. (2021) 14:343. 10.1186/s13071-021-04840-0 34187544 PMC8244189

[29] Parasitesandvectors.biomedcentral.com. Identification of tick-borne pathogens by metagenomic next-generation sequencing in Dermacentor nuttalli and Ixodes persulcatus in Inner Mongolia, China. Parasites & Vectors. Available online at: https://parasitesandvectors.biomedcentral.com/articles/10.1186/s13071-021-04740-3 (accessed March 23, 2022). 10.1186/s13071-021-04740-3 PMC8161991 34044867

[30] Murray CJ Ikuta KS Sharara F Swetschinski L Aguilar GR Gray A. Global burden of bacterial antimicrobial resistance in 2019: a systematic analysis. Lancet. (2022) 399:629–55. 10.1016/S0140-6736(21)02724-0 35065702 PMC8841637

[31] Laxminarayan R Duse A Wattal C Zaidi AKM Wertheim HFL Sumpradit N. Antibiotic resistance-the need for global solutions. Lancet Infect Dis. (2013) 13:1057–98. 10.1016/S1473-3099(13)70318-9 24252483

[32] Yong D Toleman MA Giske CG Cho HS Sundman K Lee K. Characterization of a new metallo-beta-lactamase gene, bla(NDM-1), and a novel erythromycin esterase gene carried on a unique genetic structure in Klebsiella pneumoniae sequence type 14 from India. Antimicrob Agents Chemother. (2009) 53:5046–54. 10.1128/AAC.00774-09 19770275 PMC2786356

[33] Liu YY Wang Y Walsh TR Yi LX Zhang R Spencer J. Emergence of plasmid-mediated colistin resistance mechanism MCR-1 in animals and human beings in China: a microbiological and molecular biological study. Lancet Infect Dis. (2016) 16:161–8. 10.1016/S1473-3099(15)00424-7 26603172

[34] Quan J Langelier C Kuchta A Batson J Teyssier N Lyden A. FLASH: a next-generation CRISPR diagnostic for multiplexed detection of antimicrobial resistance sequences. Nucleic Acids Res. (2019) 47:e83. 10.1093/nar/gkz418 31114866 PMC6698650

[35] Pasolli E Asnicar F Manara S Zolfo M Karcher N Armanini F. Extensive unexplored human microbiome diversity revealed by over 150,000 genomes from metagenomes spanning age, geography, and lifestyle. Cell. (2019) 176:649–62.e20. 10.1016/j.cell.2019.01.001 30661755 PMC6349461

[36] Tamburini FB Maghini D Oduaran OH Brewster R Hulley MR Sahibdeen V. Short- and long-read metagenomics of urban and rural South African gut microbiomes reveal a transitional composition and undescribed taxa. Nat Commun. (2022) 13:926. 10.1038/s41467-021-27917-x 35194028 PMC8863827

[37] Hendriksen RS Munk P Njage P van Bunnik B McNally L Lukjancenko O. Global monitoring of antimicrobial resistance based on metagenomics analyses of urban sewage. Nat Commun. (2019) 10:1124. 10.1038/s41467-019-08853-3 30850636 PMC6408512

[38] Pehrsson EC Tsukayama P Patel S Mejía-Bautista M Sosa-Soto G Navarrete KM. Interconnected microbiomes and resistomes in low-income human habitats. Nature. (2016) 533:212–6. 10.1038/nature17672 27172044 PMC4869995

[39] Manning JE Bohl JA Lay S Chea S Sovann L Sengdoeurn Y. Rapid metagenomic characterization of a case of imported COVID-19 in Cambodia. BioRxiv. (2020) 2020:968818. 10.1101/2020.03.02.968818 32511296 PMC7217139

[40] Murillo J Villegas LM Ulloa-Murillo LM Rodríguez AR. Recent trends on omics and bioinformatics approaches to study SARS-CoV-2: a bibliometric analysis and mini-review. Comput Biol Med. (2021) 128:104162. 10.1016/j.compbiomed.2020.104162 33310371 PMC7710474

[41] Xu J Wang Y Li Y Yang H Pan M Liu J. A case study of applying metagenomic sequencing in precise epidemiology for the COVID-19 pandemic — Sichuan Province, China, 2020. China CDC Wkly. (2020) 2:897–901. 10.46234/ccdcw2020.244 34594795 PMC8393149

[42] WHO. Laboratory Guidance. (2020). Available online at: https://www.who.int/emergencies/diseases/novel-coronavirus-2019/technical-guidance/laboratory-guidance (accessed February 10, 2020).

[43] Ng OW Tan YJ. Understanding bat SARS-like coronaviruses for the preparation of future coronavirus outbreaks — implications for coronavirus vaccine development. Hum Vaccines Immunother. (2017) 13:186–9. 10.1080/21645515.2016.1228500 27644155 PMC5287300

[44] Babiker A Bradley HL Stittleburg VD Ingersoll JM Key A Kraft CS. Metagenomic sequencing to detect respiratory viruses in persons under investigation for COVID-19. J Clin Microbiol. (2020) 59:e02142–20. 10.1128/JCM.02142-20 33067271 PMC7771462

[45] Brown KA Gubbay J Hopkins J Patel S Buchan SA Daneman N. S-gene target failure as a marker of variant B.1.1.7 among SARS-CoV-2 isolates in the Greater Toronto Area, December 2020 to March 2021. J Am Med Assoc. (2021) 325:2115–6. 10.1001/jama.2021.5607 33830171 PMC8033504

[46] Takashita E Kinoshita N Yamayoshi S Sakai-Tagawa Y Fujisaki S Ito M. Efficacy of antiviral agents against the SARS-CoV-2 omicron subvariant BA.2. N Engl J Med. (2022) 2022:NEJMc2201933. 10.1056/NEJMc2201933 35263535 PMC8929374

[47] Maxmen A. Omicron blindspots: why it's hard to track coronavirus variants. Nature. (2021) 600:579–579. 10.1038/d41586-021-03698-7 34916668

[48] Saha S Malaker R Sajib MSI Hasanuzzaman M Rahman H Ahmed ZB. Complete genome sequence of a novel coronavirus (SARS-CoV-2) isolate from Bangladesh. Microbiol Resour Announc. (2022) 9:e00568–20. 10.1128/MRA.00568-20 32527780 PMC7291105

[49] Shrestha R Katuwal N Adhikari N Vanaerschot M Tamrakar D Dhimal M. Whole genome sequence analysis to identify SARS-CoV-2 variant in Nepal. Kathmandu Univ Med J KUMJ. (2021) 19:137–42. 34819443

[50] Tegally H Wilkinson E Giovanetti M Iranzadeh A Fonseca V Giandhari J. Detection of a SARS-CoV-2 variant of concern in South Africa. Nature. (2021) 592:438–43. 10.1038/s41586-021-03402-9 33690265

[51] Randremanana RV Andriamandimby S Rakotondramanga JM Razanajatovo NH Mangahasimbola RT Randriambolamanantsoa TH. The COVID-19 epidemic in Madagascar: clinical description and laboratory results of the first wave, march-september 2020. Influenza Other Respir Viruses. (2021) 15:457–68. 10.1111/irv.12845 33586912 PMC8013501

[52] Faria NR Mellan TA Whittaker C Claro IM Candido DS Mishra S. Genomics and epidemiology of the P1 SARS-CoV-2 lineage in Manaus, Brazil. Science. (2021) 372:815–21. 10.1126/science.abh2644 33853970 PMC8139423

[53] GISAID. Submission Tracker Global. Available online at: https://www.gisaid.org/submission-tracker-global/ (accessed April 12, 2022).

[54] WHO. The Access to COVID-19 Tools (ACT) Accelerator. Available online at: https://www.who.int/initiatives/act-accelerator (accessed April 5, 2022).

[55] Eigbike M,. Health Research Capacity Strengthening in Low Middle-Income Countries: Current Situation Opportunities to Leverage Data for Better Coordination Greater Impact. World Health Organ. (2020). Available online at: https://www.who.int/tdr/partnerships/essence/meetings/ESSENCE-Mechanism-consultant-report.pdf?ua=1 (accessed March 29, 2022).

[56] UIS. UIS Statistics. Available online at: http://data.uis.unesco.org/ (accessed March 29, 2022).

[57] British Council,. African Network for Internationalisation of Education. Building PhD capacity in Sub-Saharan Africa. (2018). Available online at: https://www.britishcouncil.org/sites/default/files/h233_07_synthesis_report_final_web.pdf (accessed April 11, 2022).

[58] Junier T Huber M Schmutz S Kufner V Zagordi O Neuenschwander S. Viral metagenomics in the clinical realm: lessons learned from a Swiss-Wide Ring Trial. Genes. (2019) 10:655. 10.3390/genes10090655 31466373 PMC6770386

[59] De Las Rivas J Bonavides-Martínez C Campos-Laborie FJ. Bioinformatics in Latin America and SoIBio impact, a tale of spin-off and expansion around genomes and protein structures. Brief Bioinform. (2017) 20:390–7. 10.1093/bib/bbx114 28981567 PMC6433739

[60] Ras V Carvajal-López P Gopalasingam P Matimba A Chauke PA Mulder N. Challenges and considerations for delivering bioinformatics training in LMICs: perspectives from Pan-African and Latin American Bioinformatics Networks. Front Educ. (2021) 6:710971. 10.3389/feduc.2021.710971

[61] Bezuidenhout LM Leonelli S Kelly AH Rappert B. Beyond the digital divide: towards a situated approach to open data. Sci Public Policy. (2017) 44:464–75. 10.1093/scipol/scw036

[62] Hu T Chitnis N Monos D Dinh A. Next-generation sequencing technologies: an overview. Hum Immunol. (2021) 82:801–11. 10.1016/j.humimm.2021.02.012 33745759

[63] Meyer F Paarmann D D'Souza M Olson R Glass E Kubal M. The metagenomics RAST server – a public resource for the automatic phylogenetic and functional analysis of metagenomes. BMC Bioinformat. (2008) 9:386. 10.1186/1471-2105-9-386 18803844 PMC2563014

[64] Vilsker M Moosa Y Nooij S Fonseca V Ghysens Y Dumon K. Genome detective: an automated system for virus identification from high-throughput sequencing data. Bioinformatics. (2019) 35:871–3. 10.1093/bioinformatics/bty695 30124794 PMC6524403

[65] Minot SS Krumm N Greenfield NB. One codex: a sensitive and accurate data platform for genomic microbial identification. BioRxiv. (2015) 2015:e027607. 10.1101/027607

[66] Rodriguez-Tomé P. Resources at EBI. Methods Mol Biol Clifton NJ. (2000) 132:313–35. 10.1385/1-59259-192-2:313 10547844

[67] Weber N Liou D Dommer J MacMenamin P Quiñones M Misner I. Nephele: a cloud platform for simplified, standardized and reproducible microbiome data analysis. Bioinforma Oxf Engl. (2018) 34:1411–3. 10.1093/bioinformatics/btx617 29028892 PMC5905584

[68] Flygare S Simmon K Miller C Qiao Y Kennedy B Di Sera T. Taxonomer: an interactive metagenomics analysis portal for universal pathogen detection and host mRNA expression profiling. Genome Biol. (2016) 17:111. 10.1186/s13059-016-0969-1 27224977 PMC4880956

[69] Zinter MS Mayday MY Ryckman KK Jelliffe-Pawlowski LL DeRisi JL. Towards precision quantification of contamination in metagenomic sequencing experiments. Microbiome. (2019) 7:62. 10.1186/s40168-019-0678-6 30992055 PMC6469116

[70] Madden DE Webb JR Steinig EJ Currie BJ Price EP Sarovich DS. Taking the next-gen step: Comprehensive antimicrobial resistance detection from *Burkholderia pseudomallei*. eBioMedicine. (2021) 63:103152. 10.1016/j.ebiom.2020.103152 33285499 PMC7724162

[71] Lucchi NW Oberstaller J Kissinger JC Udhayakumar V. Malaria diagnostics and surveillance in the post-genomic era. Public Health Genomics. (2013) 16:37–43. 10.1159/000345607 23548716 PMC4694569

[72] Retchless AC Fox LM Maiden MCJ Smith V Harrison LH Glennie L. Toward a global genomic epidemiology of meningococcal disease. J Infect Dis. (2019) 220(Suppl.4):S266–73. 10.1093/infdis/jiz279 31671445

[73] ITU Hub,. Facts Figures 2021: 2.9 Billion People Still Offline. (2021). Available online at: https://www.itu.int/hub/2021/11/facts-and-figures-2021-2-9-billion-people-still-offline/ (accessed March 29, 2022).

[74] Cable.co.uk. Worldwide Broadband Speed League. (2021). Available online at: https://www.cable.co.uk/broadband/speed/worldwide-speed-league/ (accessed March 23, 2022).

[75] Mulder N Adebamowo CA Adebamowo SN Adebayo O Adeleye O Alibi M. Genomic research data generation, analysis and sharing – challenges in the African Setting. Data Sci J. (2017) 16:49. 10.5334/dsj-2017-049

[76] Human Rights Watch,. Shutting Down the Internet to Shut Up Critics. In: World Report. (2020). Available online at: https://www.hrw.org/world-report/2020/country-chapters/global-5 (accessed April 11, 2022).

[77] Amolo GO. The growth of high-performance computing in Africa. Comput Sci Eng. (2018) 20:21–4. 10.1109/MCSE.2018.03221926

[78] Genome.gov. The Cost of Sequencing a Human Genome. Available online at: https://www.genome.gov/about-genomics/fact-sheets/Sequencing-Human-Genome-cost (accessed July 26, 2022).

[79] Time. TIME100 Most Influential Companies 2021: Illumina. (2021). Available online at: https://time.com/collection/time100-companies/5953584/illumina/ (accessed March 24, 2022).

[80] Cost of NGS. Comparisons and Budget Guidance. Available online at: https://www.illumina.com/science/technology/next-generation-sequencing/beginners/ngs-cost.html (accessed April 5, 2022).

[81] Oxford, Nanopore Technologies,. Product Comparison. Available online at: http://nanoporetech.com/products/comparison (accessed April 5, 2022).

[82] Levenson, D,. Metagenomic Next-generation Sequencing. American Association for Clinical Chemistry. Available online at: https://www.aacc.org/cln/articles/2020/janfeb/metagenomic-next-generation-sequencing (accessed April 12, 2022).

[83] The H3Africa Consortium. Enabling the genomic revolution in Africa. Science. (2014) 344:1346–8. 10.1126/science.1251546 24948725 PMC4138491

[84] Fogarty International Center,. Fogarty Awards $5M for Bioinformatics Research Training in Africa. (2017). Available online at: https://www.fic.nih.gov:443/News/Pages/2017-h3africa-bioinformatics-training.aspx (accessed April 13, 2022).

[85] Bill Melinda Gates Foundation Global Grand Challenges. Metagenomic Next Generation Sequencing to Detect, Identify, and Characterize Pathogens. (2022). Available online at: https://gcgh.grandchallenges.org/challenge/metagenomic-next-generation-sequencing-detect-identify-and-characterize-pathogens (accessed April 13, 2022).

[86] Helmy M Awad M Mosa KA. Limited resources of genome sequencing in developing countries: challenges and solutions. Appl Transl Genomics. (2016) 9:15–9. 10.1016/j.atg.2016.03.003 27354935 PMC4911431

[87] Gordy D Tashjian RS Lee H Movassaghi M Yong WH. Domestic and international shipping of biospecimens. Methods Mol Biol Clifton NJ. (2019) 1897:433–43. 10.1007/978-1-4939-8935-5_35 30539463 PMC6777724

[88] Hasselback L Crawford J Chaluco T Rajagopal S Prosser W Watson N. Rapid diagnostic test supply chain and consumption study in Cabo Delgado, Mozambique: estimating stock shortages and identifying drivers of stock-outs. Malar J. (2014) 13:295. 10.1186/1475-2875-13-295 25086645 PMC4237853

[89] Secretariat of the Convention on Biological Diversity. Unit B. Parties to the Nagoya Protocol. (2022). Available online at: https://www.cbd.int/abs/nagoya-protocol/signatories/ (accessed April 5, 2022).

[90] Dos S. Ribeiro C, Koopmans MP, Haringhuizen GB. Threats to timely sharing of pathogen sequence data. Science. (2018) 362:404–6. 10.1126/science.aau5229 30361362

[91] Brown JR Bharucha T Breuer J. Encephalitis diagnosis using metagenomics: application of next generation sequencing for undiagnosed cases. J Infect. (2018) 76:225–40. 10.1016/j.jinf.2017.12.014 29305150 PMC7112567

[92] Carroll D Daszak P Wolfe ND Gao GF Morel CM Morzaria S. The global virome project. Science. (2018) 359:872–4. 10.1126/science.aap7463 29472471

[93] Maxmen A. Why some researchers oppose unrestricted sharing of coronavirus genome data. Nature. (2021) 593:176–7. 10.1038/d41586-021-01194-6 33953391

[94] World Health Organization. WHO Advice for International Traffic in Relation to the SARS-CoV-2 Omicron Variant (B.1.1.529). Geneva: World Health Organization (2021). Available online at: https://www.who.int/news-room/articles-detail/who-advice-for-international-traffic-in-relation-to-the-SARS-CoV-2-omicron-variant (accessed April 13, 2022).

[95] Shu Y McCauley J GISAID. Global initiative on sharing all influenza data - from vision to reality. Euro Surveill Bull Eur Sur Mal Transm Eur Commun Dis Bull. (2017) 30:22. 10.2807/1560-7917.ES.2017.22.13.30494 28382917 PMC5388101

[96] SCIENCE. Critics Decry Access, Transparency Issues With Key Trove of Coronavirus Sequences. (2022). Available online at: https://www.science.org/content/article/critics-decry-access-transparency-issues-key-trove-coronavirus-sequences (accessed April 11, 2022).

[97] Wellcome. Publishers Make Coronavirus (COVID-19) Content Freely Available and Reusable. Available online at: https://wellcome.org/press-release/publishers-make-coronavirus-covid-19-content-freely-available-and-reusable (accessed April 11, 2022).

[98] Fraser N Brierley L Dey G Polka JK Pálfy M Nanni F. The evolving role of preprints in the dissemination of COVID-19 research and their impact on the science communication landscape. PLoS Biol. (2021) 19:e3000959. 10.1371/journal.pbio.3000959 33798194 PMC8046348

[99] Kwon D. ResearchGate dealt a blow in copyright lawsuit. Nature. (2022) 603:375–6. 10.1038/d41586-022-00513-9 35246643

[100] Inzaule SC Tessema SK Kebede Y Ogwell Ouma AE Nkengasong JN. Genomic-informed pathogen surveillance in Africa: opportunities and challenges. Lancet Infect Dis. (2021) 21:e281–9. 10.1016/S1473-3099(20)30939-7 33587898 PMC7906676

[101] NIH. West African Sustainable Leadership and Innovation Training in Bioinformatics Research (WASLITBRe). Available online at: https://reporter.nih.gov/project-details/9386917 (accessed April 13, 2022).

[102] Abrudan M Matimba A Nikolic D Hughes D Argimón S Kekre M. Train-the-trainer as an effective approach to building global networks of experts in genomic surveillance of antimicrobial resistance (AMR). Clin Infect Dis Off Publ Infect Dis Soc Am. (2021) 73(Suppl.4):S283–9. 10.1093/cid/ciab770 34850831 PMC8634536

[103] Gallardo-Alba C Grüning B Serrano-Solano B. A constructivist-based proposal for bioinformatics teaching practices during lockdown. PLoS Comput Biol. (2021) 17:e1008922. 10.1371/journal.pcbi.1008922 33983931 PMC8118257

[104] THEGLOBALFUND. Development of the Global Fund Strategy (2023-2028). (2022). Available online at: https://www.theglobalfund.org/en/strategy/development-of-the-global-fund-strategy-2023-2028/ (accessed April 11, 2022).

[105] Palen J El-Sadr W Phoya A Imtiaz R Einterz R Quain E. PEPFAR, health system strengthening, and promoting sustainability and country ownership. J Acquir Immune Defic Syndr. (2012) 60:S113. 10.1097/QAI.0b013e31825d28d7 22797732

[106] Orza M Scott K Smits H Holzemer W Curran J Carpenter C. PEPFAR Implementation: Progress and Promise. Washington, DC: National Academies Press (2007).

[107] Chai JH Lee CK Lee HK Wong N Teo K Tan CS. Cost-benefit analysis of introducing next-generation sequencing (metagenomic) pathogen testing in the setting of pyrexia of unknown origin. PLoS ONE. (2018) 13:e0194648. 10.1371/journal.pone.0194648 29664913 PMC5903630

[108] Lewandowska DW Zagordi O Zbinden A Schuurmans MM Schreiber P Geissberger FD. Unbiased metagenomic sequencing complements specific routine diagnostic methods and increases chances to detect rare viral strains. Diagn Microbiol Infect Dis. (2015) 83:133–8. 10.1016/j.diagmicrobio.2015.06.017 26231254 PMC7172999

[109] FIND. NGS Capacity Mapping. (2022). Available online at: https://www.finddx.org/sequencing/ngs-capacity-mapping/ (accessed April 5, 2022).

[110] KOUNTABLE. Global Trade and Technology Platform. (2022). Available online at: https://www.kountable.com/ (accessed March 29, 2022).

[111] VIA Global Health. Shop - Global Health and Medical Supplies. (2022). Available online at: https://viaglobalhealth.com/ (accessed March 29, 2022).

[112] Ahmed AE Mpangase PT Panji S Baichoo S Souilmi Y Fadlelmola FM. Organizing and running bioinformatics hackathons within Africa: the H3ABioNet cloud computing experience. AAS Open Res. (2019) 1:9. 10.12688/aasopenres.12847.2 32382696 PMC7194140

[113] Lindmeier, C,. WHO Joins Coalition for Free Digital Access to Health Research. World Health Organization. Available online at: https://www.who.int/news/item/29-08-2019-who-joins-coalition-for-free-digital-access-to-health-research (accessed April 13, 2022).

[114] Friedrich MJ. Ethical guidelines for genomic research in Africa. J Am Med Assoc. (2018) 319:2371. 10.1001/jama.2018.7241 29922834

