# Supplementary material for: Metagenomic pathogen sequencing in resource-scarce settings: Lessons learned and the road ahead
Source: Front Epidemiol. 2022 Aug 15;2:926695. doi: 10.3389/fepid.2022.926695 (PMC9558322; doi:10.3389/fepid.2022.926695)
Supplement: Supplementary file 1 [file Data_Sheet_1.docx]

**Supplementary Data**

Supplementary Data: Search strategy to identify examples of mNGS applications for pathogen detection in LMICs. Search terms were entered in the Medline database. Abstracts of articles were screened for relevance by two authors (CY and AP) and full text of relevant articles were reviewed. Bibliographies of relevant articles were examined to identify additional studies. Search was last updated on July 26, 2022.

("Afghanistan"[Mesh] OR "Burundi"[Mesh] OR "Burkina Faso"[Mesh] OR "Central African Republic"[Mesh] OR "Democratic Republic of the Congo"[Mesh] OR "Eritrea"[Mesh] OR "Ethiopia"[Mesh] OR "Guinea"[Mesh] OR "Gambia"[Mesh] OR "Guinea-Bissau"[Mesh] OR "Liberia"[Mesh] OR "Madagascar"[Mesh] OR "Mali"[Mesh] OR "Mozambique"[Mesh] OR "Malawi"[Mesh] OR "Niger"[Mesh] OR "Democratic People's Republic of Korea"[Mesh] OR "Rwanda"[Mesh] OR "Sudan"[Mesh] OR "Sierra Leone"[Mesh] OR "Somalia"[Mesh] OR "South Sudan"[Mesh] OR "Syria"[Mesh] OR "Chad"[Mesh] OR "Togo"[Mesh] OR "Uganda"[Mesh] OR "Yemen"[Mesh] OR "Zambia"[Mesh] OR "Angola"[Mesh] OR "Benin"[Mesh] OR "Bangladesh"[Mesh] OR "Bolivia"[Mesh] OR "Bhutan"[Mesh] OR "Cote d'Ivoire"[Mesh] OR "Cameroon"[Mesh] OR "Congo"[Mesh] OR "Comoros"[Mesh] OR "Cabo Verde"[Mesh] OR "Djibouti"[Mesh] OR "Algeria"[Mesh] OR "Egypt"[Mesh] OR "Micronesia"[Mesh] OR "Ghana"[Mesh] OR "Honduras"[Mesh] OR "Haiti"[Mesh] OR "Indonesia"[Mesh] OR "India"[Mesh] OR "Iran"[Mesh] OR "Kenya"[Mesh] OR "Kyrgyzstan"[Mesh] OR "Cambodia"[Mesh] OR "Laos"[Mesh] OR "Lebanon"[Mesh] OR "Sri Lanka"[Mesh] OR "Lesotho"[Mesh] OR "Morocco"[Mesh] OR "Myanmar"[Mesh] OR "Mongolia"[Mesh] OR "Mauritania"[Mesh] OR "Nigeria"[Mesh] OR "Nicaragua"[Mesh] OR "Nepal"[Mesh] OR "Pakistan"[Mesh] OR "Philippines"[Mesh] OR "Papua New Guinea"[Mesh] OR "Senegal"[Mesh] OR "Melanesia"[Mesh] OR "El Salvador"[Mesh] OR "Sao Tome and Principe"[Mesh] OR "Eswatini"[Mesh] OR "Tajikistan"[Mesh] OR "Timor-Leste"[Mesh] OR "Tunisia"[Mesh] OR "Tanzania"[Mesh] OR "Ukraine"[Mesh] OR "Uzbekistan"[Mesh] OR "Vietnam"[Mesh] OR "Samoa"[Mesh] OR "Zimbabwe"[Mesh] OR "Albania"[Mesh] OR "Argentina"[Mesh] OR "Armenia"[Mesh] OR "American Samoa"[Mesh] OR "Azerbaijan"[Mesh] OR "Bulgaria"[Mesh] OR "Bosnia and Herzegovina"[Mesh] OR "Republic of Belarus"[Mesh] OR "Belize"[Mesh] OR "Brazil"[Mesh] OR "Botswana"[Mesh] OR "China"[Mesh] OR "Colombia"[Mesh] OR "Costa Rica"[Mesh] OR "Cuba"[Mesh] OR "Dominica"[Mesh] OR "Dominican Republic"[Mesh] OR "Ecuador"[Mesh] OR "Fiji"[Mesh] OR "Gabon"[Mesh] OR "Georgia"[Mesh] OR "Equatorial Guinea"[Mesh] OR "Grenada"[Mesh] OR "Guatemala"[Mesh] OR "Guyana"[Mesh] OR "Iraq"[Mesh] OR "Jamaica"[Mesh] OR "Jordan"[Mesh] OR "Kazakhstan"[Mesh] OR "Libya"[Mesh] OR "Saint Lucia"[Mesh] OR "Moldova"[Mesh] OR "Mexico"[Mesh] OR "Republic of North Macedonia"[Mesh] OR "Montenegro"[Mesh] OR "Mauritius"[Mesh] OR "Malaysia"[Mesh] OR "Namibia"[Mesh] OR "Peru"[Mesh] OR "Palau"[Mesh] OR "Paraguay"[Mesh] OR "Russia"[Mesh] OR "Serbia"[Mesh] OR "Suriname"[Mesh] OR "Thailand"[Mesh] OR "Turkmenistan"[Mesh] OR "Tonga"[Mesh] OR "Turkey"[Mesh] OR "Saint Vincent and the Grenadines"[Mesh] OR "Kosovo"[Mesh] OR "South Africa"[Mesh] OR "Developing countries" [Mesh]) AND ("Communicable Diseases"[Mesh] OR "Infectious Disease Medicine"[Mesh] OR "Disease Transmission, Infectious"[Mesh] OR "Disease Notification"[Mesh] OR "Disease Outbreaks"[Mesh] OR "Disease Reservoirs"[Mesh] OR "Zoonoses"[Mesh]) AND ("High-Throughput Nucleotide Sequencing"[Mesh] OR "Nanopore Sequencing"[Mesh])

Supplementary Table: Number of SARS-CoV-2 sequences shared to GISAID as of July 26^th^, 2022 by country/territory and economic status, full version. Data source: https://www.gisaid.org/submission-tracker-global/ (accessed July 26, 2022). Country/territory income classification were determined using World Bank definitions: https://datahelpdesk.worldbank.org/knowledgebase/articles/906519-world-bank-country-and-lending-groups (*World Bank classification not available for 21 countries).

| **Country / Territory** | | **Number of Sequences (% of Total)** |
| --- | --- | --- |
| **High-income economies** | | **11,124,200 (92.8)** |
|  | United States of America | 3,734,418 |
|  | United Kingdom | 2,764,684 |
|  | Germany | 715,173 |
|  | Denmark | 542,561 |
|  | Canada | 404,512 |
|  | France | 398,007 |
|  | Japan | 318,929 |
|  | Sweden | 198,749 |
|  | Austria | 148,351 |
|  | Spain | 146,814 |
|  | Switzerland | 145,417 |
|  | Belgium | 138,241 |
|  | Italy | 136,867 |
|  | Australia | 133,053 |
|  | Netherlands | 130,113 |
|  | Israel | 112,418 |
|  | Turkey | 94,646 |
|  | Poland | 83,766 |
|  | Ireland | 81,602 |
|  | Slovenia | 70,776 |
|  | Norway | 69,008 |
|  | South Korea | 67,541 |
|  | Czech Republic | 45,507 |
|  | Lithuania | 40,286 |
|  | Luxembourg | 40,031 |
|  | Portugal | 39,594 |
|  | Slovakia | 38,291 |
|  | Finland | 35,160 |
|  | Croatia | 34,470 |
|  | Chile | 31,579 |
|  | Singapore | 20,305 |
|  | Latvia | 18,283 |
|  | Greece | 18,258 |
|  | New Zealand | 16,429 |
|  | Romania | 15,495 |
|  | Estonia | 12,939 |
|  | Puerto Rico | 11,262 |
|  | Hong Kong | 10,582 |
|  | Iceland | 10,331 |
|  | Panama | 5,110 |
|  | Qatar | 4,943 |
|  | Aruba | 3,379 |
|  | Trinidad and Tobago | 3,161 |
|  | Northern Mariana Islands | 3,025 |
|  | Gibraltar | 2,835 |
|  | United Arab Emirates | 2,615 |
|  | Brunei | 2,601 |
|  | Sint Maarten | 2,531 |
|  | Bahrain | 2,271 |
|  | U.S. Virgin Islands | 2,130 |
|  | Curaçao | 1,897 |
|  | Seychelles | 1,413 |
|  | Liechtenstein | 1,390 |
|  | Cyprus | 1,382 |
|  | Saudi Arabia | 1,344 |
|  | Oman | 1,018 |
|  | Uruguay | 942 |
|  | Kuwait | 895 |
|  | Malta | 893 |
|  | Guam | 830 |
|  | Hungary | 549 |
|  | Taiwan | 519 |
|  | Saint Martin | 315 |
|  | Andorra | 273 |
|  | The Bahamas | 263 |
|  | Antigua and Barbuda | 219 |
|  | British Virgin Islands | 195 |
|  | Barbados | 140 |
|  | Bermuda | 134 |
|  | French Polynesia | 110 |
|  | Cayman Islands | 101 |
|  | Monaco | 97 |
|  | Saint Kitts and Nevis | 74 |
|  | Turks and Caicos Islands | 72 |
|  | Faroe Islands | 42 |
|  | Japan | 35 |
|  | New Caledonia | 9 |
|  | Greenland | 0 |
|  | Guernsey | 0 |
|  | Isle of Man | 0 |
|  | Jersey | 0 |
|  | Nauru | 0 |
|  | San Marino | 0 |
|  | United States Virgin Islands | 0 |
| **Upper-middle-income economies** | | **482,200 (4.0)** |
|  | Brazil | 163,753 |
|  | Mexico | 68,623 |
|  | South Africa | 43,888 |
|  | Thailand | 27,641 |
|  | Peru | 25,677 |
|  | Malaysia | 21,802 |
|  | Colombia | 21,177 |
|  | Argentina | 20,091 |
|  | Russia | 19,528 |
|  | Bulgaria | 18,271 |
|  | Ecuador | 6,183 |
|  | Costa Rica | 5,528 |
|  | Botswana | 4,291 |
|  | Mauritius | 3,051 |
|  | China | 2,515 |
|  | Guatemala | 2,204 |
|  | Paraguay | 2,104 |
|  | Georgia | 2,020 |
|  | Jamaica | 1,958 |
|  | Namibia | 1,615 |
|  | Cuba | 1,521 |
|  | Bosnia and Herzegovina | 1,510 |
|  | Kazakhstan | 1,498 |
|  | Jordan | 1,486 |
|  | Kosovo | 1,452 |
|  | Serbia | 1,383 |
|  | Dominican Republic | 1,320 |
|  | Maldives | 1,294 |
|  | Suriname | 1,124 |
|  | Iraq | 1,107 |
|  | Gabon | 970 |
|  | North Macedonia | 928 |
|  | Belize | 842 |
|  | Montenegro | 750 |
|  | Moldova | 605 |
|  | Fiji | 531 |
|  | Belarus | 523 |
|  | Saint Vincent and the Grenadines | 217 |
|  | Equatorial Guinea | 213 |
|  | Armenia | 192 |
|  | Saint Lucia | 184 |
|  | Azerbaijan | 151 |
|  | Guyana | 145 |
|  | American Samoa | 72 |
|  | Libya | 69 |
|  | Albania | 58 |
|  | Grenada | 58 |
|  | Dominica | 39 |
|  | Palau | 35 |
|  | Marshall Islands | 3 |
|  | Tonga | 0 |
|  | Turkmenistan | 0 |
|  | Tuvalu | 0 |
| **Low- and Lower-middle-income economies** | | **357,373 (3.0)** |
|  | India | 212,558 |
|  | Indonesia | 29,609 |
|  | Philippines | 21,125 |
|  | Kenya | 10,983 |
|  | Bangladesh | 7,197 |
|  | Nigeria | 6,823 |
|  | Vietnam | 5,586 |
|  | Papua New Guinea | 4,382 |
|  | Senegal | 3,909 |
|  | Ghana | 3,891 |
|  | Pakistan | 3,540 |
|  | Sri Lanka | 3,451 |
|  | Cambodia | 3,253 |
|  | Nepal | 3,193 |
|  | Iran | 2,392 |
|  | Egypt | 2,388 |
|  | Lebanon | 2,294 |
|  | Zambia | 1,757 |
|  | Angola | 1,282 |
|  | Uganda | 1,278 |
|  | Tunisia | 1,247 |
|  | Gambia | 1,240 |
|  | Malawi | 1,203 |
|  | Mozambique | 1,193 |
|  | Democratic Republic of the Congo | 1,191 |
|  | Cameroon | 1,171 |
|  | Mongolia | 1,070 |
|  | Morocco | 993 |
|  | Ukraine | 988 |
|  | eSwatini | 976 |
|  | Zimbabwe | 930 |
|  | Rwanda | 916 |
|  | Madagascar | 879 |
|  | Benin | 861 |
|  | Togo | 811 |
|  | Côte d'Ivoire | 758 |
|  | Djibouti | 716 |
|  | Burkina Faso | 665 |
|  | Ethiopia | 628 |
|  | Guinea | 623 |
|  | Republic of the Congo | 614 |
|  | Venezuela | 590 |
|  | Nicaragua | 566 |
|  | El Salvador | 522 |
|  | Cabo Verde | 480 |
|  | Laos | 372 |
|  | Timor-Leste | 356 |
|  | Bolivia | 345 |
|  | Niger | 344 |
|  | Kyrgyzstan | 330 |
|  | Algeria | 329 |
|  | Solomon Islands | 246 |
|  | Honduras | 231 |
|  | Lesotho | 219 |
|  | Sudan | 199 |
|  | Haiti | 186 |
|  | South Sudan | 170 |
|  | Burundi | 158 |
|  | Myanmar | 137 |
|  | West Bank and Gaza | 133 |
|  | Central African Republic | 110 |
|  | Liberia | 110 |
|  | Afghanistan | 103 |
|  | Uzbekistan | 90 |
|  | Syria | 89 |
|  | Mali | 74 |
|  | Sierra Leone | 61 |
|  | Mauritania | 51 |
|  | Chad | 49 |
|  | Guinea-Bissau | 48 |
|  | Somalia | 35 |
|  | Comoros | 34 |
|  | Kiribati | 30 |
|  | Tanzania | 11 |
|  | Vanuatu | 1 |
|  | Eritrea | 0 |
|  | North Korea | 0 |
|  | Yemen | 0 |
|  | Bhutan | 0 |
|  | Micronesia | 0 |
|  | Samoa | 0 |
|  | Tajikistan | 0 |
| **Countries/territories without World Bank classification of economic group*** | | **22,550 (0.2)** |
|  | Réunion | 14,493 |
|  | Martinique | 2,401 |
|  | French Guiana | 1,876 |
|  | Bonaire | 1,632 |
|  | Mayotte | 1,037 |
|  | Guadeloupe | 960 |
|  | Anguilla | 85 |
|  | Montserrat | 28 |
|  | Saint Barthélemy | 14 |
|  | Sao Tome and Principe | 10 |
|  | Wallis and Futuna Islands | 10 |
|  | Sint Eustatius | 4 |
|  | Cook Islands | 0 |
|  | Falkland Islands (Malvinas) | 0 |
|  | Holy See | 0 |
|  | Saint Helena | 0 |
|  | Tokelau | 0 |
|  | Niue | 0 |
|  | Pitcairn Islands | 0 |
|  | Saint Pierre and Miquelon | 0 |
|  | Saba | 0 |
